# Validity study of educational technology on gynecological high dose rate (HDR) brachytherapy

**DOI:** 10.1590/0034-7167-2022-0232

**Published:** 2023-04-14

**Authors:** Meiriane Lopes Ximenes, Mariana Alves Firmeza, Andrea Bezerra Rodrigues, Maria Isis Freire de Aguiar, Gabriela Lacerda Souza, Georgia de Sousa Serpa, Patrícia Peres de Oliveira

**Affiliations:** I Universidade Federal do Ceará. Fortaleza, Ceará, Brazil; II Universidade Federal de São João del-Rei. Divinópolis, Minas Gerais, Brazil

**Keywords:** Brachytherapy, Educational Technology, Validation Study, Medical Oncology, Genital Neoplasms, Female, Braquiterapia, Tecnología Educacional, Estudio de Validación, Oncología Médica, Neoplasias de los Genitales Femeninos, Braquiterapia, Tecnologia Educacional, Estudo de Validação, Oncologia, Neoplasias dos Genitais Femininos

## Abstract

**Objective:**

to construct and validate an educational booklet on high dose rate gynecological brachytherapy (HDR) for women with gynecologic cancer.

**Methods:**

a methodological study, with the construction and validity of a booklet based on the Doak, Doak and Root theoretical-methodological framework. Content and appearance validity was guided by the Delphi technique, by 11 judges, selected using Jasper’s criteria. Afterwards, clinical validity was carried out with the target population.

**Results:**

the booklet, built from evidence from an integrative review, validated with judges, obtained an overall CVI of 0.98. After clinical validity with 27 women, it presents 24 sheets with illustrations produced by a graphic designer, subdivided into topics: gynecological system anatomy and gynecological cancer epidemiology, gynecological brachytherapy definition, therapeutic steps, approach to side effects and management, and two pages for notes.

**Conclusions:**

the booklet has validity for use in HDR gynecological brachytherapy treatment.

## RESUMO

**Objetivo:**

construir e validar uma cartilha educativa sobre braquiterapia ginecológica de alta taxa de dose (*high dose radiation*-HDR) para mulheres com câncer ginecológico.

**Métodos:**

estudo metodológico, com construção e validação de uma cartilha embasada no referencial teórico-metodológico de Doak, Doak e Root. A validação de conteúdo e aparência norteou-se pela técnica Delphi, por 11 juízes, selecionados através dos critérios de Jasper. Após, realizou-se a validação clínica com a população-alvo.

**Resultados:**

a cartilha, construída a partir de evidências de uma revisão integrativa, validada com juízes, obteve IVC global de 0,98. Após validação clínica com 27 mulheres, apresenta 24 folhas, com ilustrações produzidas por designer gráfico, subdividida em tópicos: anatomia do sistema ginecológico e epidemiologia dos cânceres ginecológicos, definição de braquiterapia ginecológica, etapas terapêuticas, abordagem dos efeitos colaterais e manejo, e duas páginas destinadas para anotações.

**Conclusões:**

a cartilha possui validação para uso no tratamento de braquiterapia ginecológica de HDR.

## RESUMEN

**Objetivo:**

construir y validar un folleto educativo sobre braquiterapia ginecológica de alta tasa de dosis (radiación de alta dosis-HDR) para mujeres con cáncer ginecológico.

**Métodos:**

estudio metodológico, con la construcción y validación de un cuadernillo basado en el marco teórico-metodológico de Doak, Doak y Root. La validación de contenido y apariencia fue guiada por la técnica Delphi, por 11 jueces, seleccionados según los criterios de Jasper. Posteriormente se realizó la validación clínica con la población diana.

**Resultados:**

la cartilla, construida a partir de evidencias de revisión integradora, validada con jueces, obtuvo un IVC global de 0,98. Después de la validación clínica con 27 mujeres, presenta 24 fichas, con ilustraciones realizadas por un diseñador gráfico, subdivididas en temas: anatomía del sistema ginecológico y epidemiología de los cánceres ginecológicos, definición de braquiterapia ginecológica, pasos terapéuticos, abordaje de los efectos secundarios y manejo, y dos páginas para notas.

**Conclusiones:**

la cartilla tiene validación para su uso en el tratamiento de braquiterapia ginecológica de la HDR.

## INTRODUCTION

Among the treatment options for cancer patients is radiotherapy (RT), which involves teletherapy, in which the radiation source is positioned far from the tumor, and brachytherapy (BT), when the source is in direct contact with the tumor. The latter unfolds in two modalities: low dose rate (LDR) and high dose rate (HDR)^(1-3)^.

HDR BT is widely used in the treatment of gynecological cancers, including vagina, vulva, ovary and cervix. It is noteworthy that the latter ranks 4th place among the most frequent types of cancers in females in Brazil^(4)^ and, in its treatment, depending on the staging, can be used, often concomitantly, surgery, chemotherapy, teletherapy, especially HDR BT. This therapy has great importance for cervical cancer prognosis, because it is more directed to the treatment site, allowing the use of high radiation concentrations^(5)^.

The BT process is performed on an outpatient basis, consisting of several stages, and each one is developed by a professional from the multidisciplinary team, especially nurses^(5)^. In nursing consultation, there is guidance about the treatment, the importance of attendance, since they are around three to four sessions, as well as their possible side effects and measures to prevent or reduce such effects^(6)^. Knowing that the treatment can result in diarrhea, lymphedema, urinary symptoms, bleeding and vaginal stenosis as adverse effects, health teams must to pay attention to the orientation towards them, mainly because it is an outpatient treatment in which patients present them at home^(7)^.

In order to improve the health education process, educational technologies can be used as teaching resources, with recognized impact on effectiveness, provided they have good quality and suitable content, to facilitate the understanding of information^(8)^.

The use of educational technologies shows promising results in the most diverse specialties, with reduction of side effects and complications^(9-11)^. Moreover, the provision of guidance has a significant role in reducing anxiety and insecurity, feelings that can be experienced throughout treatment^(6)^.

Based on these premises, the purpose of this study was to build and validate a booklet on HDR gynecological BT, considering the participation of judges and women with gynecological cancer who undergo this treatment. It is noteworthy that the project is part of a multi-method study, in which this was the first stage. A randomized clinical trial is underway with the use of a previously validated booklet to assess the side effects presented, patients’ attendance to treatment, as well as the level of anxiety.

Considering the importance of using educational technologies in the professional practice of nurses and the lack of technologies aimed at assisting women undergoing HDR gynecological BT, it is considered that this educational booklet, after being validated, brings great contributions to the integral and effective health care of patients affected by gynecological cancer.

## OBJECTIVE

To construct and validate an educational booklet on HDR gynecological BT for patients with gynecologic cancer.

## METHODS

### Ethical aspects

According to the criteria established by Resolution 466/2012, the study was submitted to the Research Ethics Committee of the *Escola de Saúde Pública do Estado do Ceará* and was posted on the *Platforma Brasil* for ethical appreciation.

In the appearance and content validity phase, all judges were informed about the study objectives, and agreed to participate through the Informed Consent Form (ICF). In the clinical validity phase, participants able to the clinical validity process were informed about the study and received the ICF signed in two copies, one with the participant and the other with the researchers. As informed in the ICF, anonymity was guaranteed, with confidentiality, privacy and image protection being ensured.

### Theoretical-methodological framework

The educational booklet (EB) construction was supported by the concepts proposed by Doak, Doak and Root about the learning process of patients with low literacy skills, who claim that our learning styles are based on the five senses, touch, smell, hearing, sight and taste, predominating the use of a learning style, in many cases, vision. Thus, complex concepts can be more easily understood through visual presentations, including in the health education process^(12)^.

Some guidelines are essential for the planning steps of building materials: defining the target audience; limit and define learning objectives, which must be compatible with the actions and behaviors that the educational intervention is intended to achieve; in writing, the active voice should be used, as it makes reading easier and moves the reader to take action; use common vocabulary words, avoiding technical terms that make understanding difficult, and, if one use them, it is necessary to exemplify so that everyone can understand; write short sentences, making it easier to read and understand; include interactions that can make guidance easier to learn and remember, as well as revisions; finally, testing to ensure quality assurance^(12)^.

### Study design

This is a methodological study to build a validity EB, with EC judges and clinical validity, guided by the Consolidated Criteria for Reporting Qualitative Research (COREQ), made available by the EQUATOR Network, to guide the description of qualitative studies^(13)^.

### Methodological procedures

The study was carried out in three stages: EB construction; EB content and appearance validity by judges; EB clinical validity.

For the construction of this booklet, initially, an integrative literature review was carried out, in order to scientifically support the EB elaboration. The integrative review was guided by a methodological course, composed of six distinct steps, namely: theme identification and hypothesis or research question selection for the integrative review elaboration; establishment of criteria for inclusion and exclusion of studies/sampling or literature search; definition of the information to be extracted from the selected studies/study categorization; assessment of studies included in the integrative review; interpretation of results; and presentation of knowledge review/synthesis^(14)^. Thus, the guiding question of this integrative review to achieve the proposed objective was: what empirical indicators and guidelines should be included in an EB that systematizes nursing care for women who will undergo HDR gynecological BT? A search was performed in the Medical Literature Analysis and Retrieval System Online (MEDLINE), Latin American Literature in Health Sciences (LILACS), Scientific Electronic Library Online (SciELO), Nursing Database (BDENF), Cochrane Library and The Cumulative Index to Nursing and Allied Health Literature (CINAHL). Moreover, searches were carried out on websites of national and international RT associations recognized as relevant in oncology and RT. They were: The Radiation Therapy Oncology Group (RTOG), American Society of Clinical Oncology (ASCO), American Society for Therapeutic Radiology and Oncology (ASTRO), Oncology Nursing Society (ONS) and *Instituto Nacional de Câncer José Alencar Gomes da Silva* (INCA). To search for scientific publications, controlled descriptors related to the topic were used, such as “Uterine cervical”, “neoplasms”, “Endometrial cancer”, “Radiotherapy”, “Brachytherapy”, “Health education” and “Nursing care”, also in Portuguese and Spanish.

We included articles published in full, in Portuguese, English or Spanish, searched from the descriptors as contained in the Health Sciences Descriptors (DeCS) and Medical Subject (MeSH).

We excluded review studies and repeated studies in the databases. On the association’s websites, the search was made for keywords brachytherapy, uterine cervical neoplasms, endometrial cancer, radiodermatitis, dyspareunia, vaginal discharge, vaginal dryness, vaginal bleeding, vaginal inflammation, vaginal pain, narrowing and vaginismus.

Thus, the booklet was elaborated, consisting of the introduction of possible side effects, including their management and prevention, and the importance of treatment attendance. The illustrations were developed exclusively for the booklet through the software Clip Studio Paint.

Regarding EB content and appearance validity by judges, we used the Delphi^(15)^, which seeks to facilitate and improve decision-making made by a group of experts, in two assessment rounds, the first being carried out by eleven judges, and the second, by nine judges, selected through Jasper’s criteria^(16)^. For the judges, the validity instrument of the educational technology and the EB were sent via Google Forms. It is noteworthy that the validity instrument is divided into two parts: the first contains the judge characterization data; and the second contains instructions for filling and the items to be assessed (objectives, structure, presentation, relevance and content itself).

Finally, the third step involved the clinical validity with the target audience of women for EB application, i.e., women with gynecological cancers undergoing HDR gynecological BT, developed in October and November 2020. To compose the sample of patients, literate women were included, aged 18 years or over, with the cognitive ability to follow the guidelines given in the EB, through the application of the Mini Mental State Examination (MMSE). Women with mental disabilities were excluded.

Participants answered the MMSE, a sociodemographic questionnaire and instrument built based on the Suitability Assessment of Materials^(12)^, with assessment of content, writing, illustrations and motivation for learning. The degree of relevance was measured using a Likert-type scale with the response options “Irrelevant”, “Not very relevant”, “Really relevant” and “Very relevant”.

### Study setting

The study was completed in November 2020, with EB clinical validity. The pilot study for EB validity was developed in the RT service of the Integrated Regional Center for Oncology (CRIO), enabled by the Ministry of Health as a High Complexity Unit in Oncology (Unacon), which makes about 100 monthly applications of HDR gynecological BT sessions. In the service, women with the most diverse ages, wholly or partially hysterectomized or not hysterectomized are assisted.

### Data source

The judges who participated in the validity process were selected by snowball sampling, which is used when the population is composed of people with characteristics that are difficult to find. People with the desired profile were identified by consulting the resumes, made available by the *Plataforma Lattes* of the Brazilian National Council for Scientific and Technological Development (CNPq - *Conselho Nacional de Desenvolvimento Científico e Tecnológico*) portal, and indicated by the main researcher, who has been active in the area for over 20 years. They were invited to participate in the study and to appoint another expert/judge, and so on.

Considering that, for a pilot study, an amount of 10% is effective^(17)^, the number of women served monthly is equal to 100 and there would be a need for at least 10 women. However, aiming at greater reliability, a higher number was reached, totaling 27 women.

### Data collection and organization

For the EB content and appearance validity, an instrument was used that contains the evaluator’s personal, professional and work identification, in addition to questions related to objectives/purposes, orientation structure and presentation, relevance and, finally, recommendations and suggestions about the EB. Each question could be answered with the following items: “Not suitable”, worth 1; “Partially suitable”, worth 2; “Suitable”, worth 3; and “Totally suitable”, worth 4. According to this score, the Content Validity Index (CVI)^(18)^ was calculated.

In the clinical validity stage, an instrument was used to assess the aspects of attractiveness, persuasion, self-efficacy, understanding, clarity and degree of relevance.

### Data analysis

Data referring to the judges’ sociodemographic profile and participants’ sociodemographic and clinical profile were analyzed and presented, through descriptive statistics, through the Statistical Package for the Social Sciences (SPSS), version 2.2.

In the analysis of judges’ opinions on issues related to EB, the mean CVI of the items considered relevant was calculated, and a CVI equal to or greater than 0.80 was adopted, i.e., equivalent to 80% of agreement among judges, which is considered optimal, and those who did not reach this value were discarded. Furthermore, all the suggestions made by the judges were considered. The CVI was calculated by summing the number of responses marked by “3” or “4” by the judges, divided by the total number of responses^(18)^.

With regard to the assessment instruments used in the booklet clinical validity, data were tabulated and analyzed using descriptive statistics.

## RESULTS

Validity with judges occurred in two assessment rounds.

### Content and appearance validity in the first round assessment

The elaboration of the first version of the educational technology was entitled “Cervical cancer and gynecological BT (HDR)” (*Câncer de colo de útero e BT ginecológica (HDR)*), organized into topics, namely: what is gynecological brachytherapy (HDR)?; care before the brachytherapy session (HDR); care during the brachytherapy session (HDR); care after the brachytherapy session (HDR); presentation of possible side effects and related care; and bibliography.

The text was written using Times New Roman font, size 24 for the information, 30 in the subtitles and 36 for the cover title, and for the latter two it was also used bold. For the information in which more emphasis was needed, the use of text boxes was used, with letter size 26, bold.

Content and appearance validity, which was performed by 11 judges from different regions of the country, one from Ceará, one from Minas Gerais, five from São Paulo, three from Bahia and one from Rio de Janeiro. As for performance, nine were nurses, one was a nurse and professor, and one was a physician. As for sex, nine belonged to the female and two to the male.

A relevant portion was masters (63.6%), followed by oncology experts (45.5%), and oncology experts in the residency modality (36.4%). The time of work of judges in the oncology area was between 20 and 30 years (45.5%) and between 5 and 10 years (45.5%). Regarding the professional practice area, oncology care (63.6%) and teaching (36.3%) prevailed. Regarding the publication of research, judges who published articles involving the theme of cancer (72.7%), RT (45.5%), educational technology (36%) and instrument validity (36%) predominated.

The result obtained from the CVI in the first Delphi round, regarding the objective item, was equal to 0.72, in structure and presentation, 0.72, and in relevance, 0.84. The overall CVI scored 0.76, not reaching the percentage of 80%, changes suggested by the judges were made and the second round started.

### Content and appearance validity in the second round assessment

The educational technology was resubmitted to the same eleven judges who participated in the first round. Of these, 9 made their contribution, eight nurses and one physician, eight females and one male. A relevant portion were masters (66.7%), followed by experts (44.4%) and residency experts (33.3%). As for the area of expertise, oncology care prevailed (77.7%), followed by teaching (22.2%). Regarding the publication of research, judges who published articles on cancer (77.8%), RT (44.4%), educational technology (36%) and instrument validity (44.4%) predominated.

The result obtained from the CVI regarding the objective item was equal to 1.00, in structure and presentation, 0.95, and in relevance, 1.00. The overall CVI scored 0.98 (98%), assuring the educational technology content and appearance validity for use in the target population.

Regarding the structure and presentation, one judge suggested removing the light blue background from the booklet, but this suggestion was not accepted, as the other judges considered it totally suitable, and in the theoretical framework adopted by Doak, Doak and Root^(12)^, the visual presentations facilitate the teaching-learning process.

Table 1 shows the CVIs in the two rounds.

**Chart 1:** Suggestions provided by judges in the educational booklet validity process

Suggestions regarding content	Situation
Replace the phrase “Putting radiation inside the body” with “The applicator is placed very close to the tumor through the vagina. Thus, radiation is directed towards the tumor, killing the malignant cells and reaching a few healthy cells.”	Accepted
Differentiate information for hysterectomized and non-hysterectomized patients and emphasize the importance of treatment attendance.	Accepted
Add as guidance the use of vaginal showers with chamomile tea.	Not accepted, because no evidence was identified in the scientific literature for the use of such practice. It is noteworthy that this suggestion was given by only one judge.
**Suggestions regarding presentation and structure**	**Situation**
Remove table of contents on page 2 of the booklet.	Accepted
Remove dialog bubbles in illustrations.	Accepted
Remove upper case from the body of the text.	Accepted
Standardize the illustration representing the health professional from beginning to end of the booklet.	Accepted
Place an illustration of the applicator, and use softer background tones.	Accepted
Add a page to the end of the annotation booklet.	Accepted

**Table 1:** Content Validity Index value presentation in the two assessment rounds

Items	CVI per item Round I	CVI per item Round I
As for the objectives	0.72	1.00
1) Does the educational booklet address the needs of women with cancer undergoing high dose rate gynecological radiotherapy (HDR)?	0.73	1.00
2) Does it provide relevant information and guidance, contributing to the health education process for women undergoing high dose rate gynecological radiotherapy?	0.90	1.00
3) Is it effective for maintaining self-care at home by women undergoing high dose rate gynecological radiotherapy?	0.82	1.00
4) Does it have the ability to promote changes in behavior and attitude in women undergoing high dose rate gynecological radiotherapy?	0.90	1.00
5) Can this booklet circulate in the scientific field in oncology?	0.36	1.00
6) Can this booklet be implemented in the daily clinical practice of nurses working in radiotherapy outpatient clinics?	0.64	1.00
As for structure and presentation	0.72	0.95
1) Is the information presented clearly and objectively?	0.73	1.00
2) Is the information presented scientifically correct?	0.55	1.00
3) Does the booklet present a logical sequence?	0.73	1.00
4) Is the language used understandable at all sociocultural levels?	0.45	0.88
5) Is the information written in the booklet well structured in terms of grammar and spelling?	1.00	0.77
6) Is the type and font size shown on the booklet suitable?	0.82	1.00
7) Is the size of the graphic elements (caption letters and photos) that appear in the booklet suitable?	0.73	1.00
8) Are the esthetic elements of the image (color, space, proportion) of the booklet suitable and understandable?	0.73	0.88
9) Is the written language present in the booklet engaging?	0.82	1.00
10) Does the booklet include elements that reinforce the most relevant information for the guidance of women undergoing high dose rate gynecological radiotherapy?	0.64	0.88
11) Do the images portray what you really want to convey in terms of information?	0.64	1.00
12) Is the booklet original?	0.82	1.00
13) Is the number of pages suitable?	0.73	1.00
As for relevance	0.84	1.00
1) Is the booklet effective when it is proposed that women undergoing high dose rate gynecological radiotherapy acquire knowledge about this treatment modality?	0.73	1.00
2) Is the booklet effective when it is proposed that women undergoing high dose rate gynecological radiotherapy acquire knowledge about the management of side effects of this treatment modality?	0.73	1.00
3) Is the booklet effective when it proposes to promote the attendance of women undergoing high dose rate gynecological radiotherapy with this treatment modality?	0.82	1.00
4) Is the instrument relevant for the guidance of women undergoing high dose rate gynecological radiotherapy?	0.90	1.00
5) Is the instrument relevant to the care provided by nurses to women undergoing high dose rate gynecological radiotherapy?	0.90	1.00
6) Does this booklet allow the transfer of relevant information about high dose rate gynecological brachytherapy between the nurse and the woman undergoing this treatment modality?	1.00	1.00
Overall CVI	0.76	0.98

### Clinical validity

Of the women interviewed, 22.2% were between 20 and 39 years old, 66.7% between 40 and 59 years old, 11.1% over 60 years old. Most were married or single (37% and 33.3%, respectively). Most had cervical cancer (59.3%). As for education, 40.7% had incomplete elementary school, 29.6% had completed high school and 29.6% had higher education

All of them were undergoing concomitant hormone therapy and teletherapy, 22.2% were undergoing chemotherapy. Regarding comorbidities, 22.2% had hypertension and 14.8% had diabetes.

Also, 37% of them had an active sex life, and of these, 11.1% had pain during the sexual act, and 7.4%, bleeding.

When answering about the EB clarity and attractiveness, 100% considered the booklet to be clear and attractive. Regarding relevance, 81.4% considered it very relevant, and 18.5%, really relevant.

### Final version of the educational technology

To identify the booklet, we use the International Standard Book Number (ISBN), represented by Code 978-65-00-36471-2.

**Figure 1:**
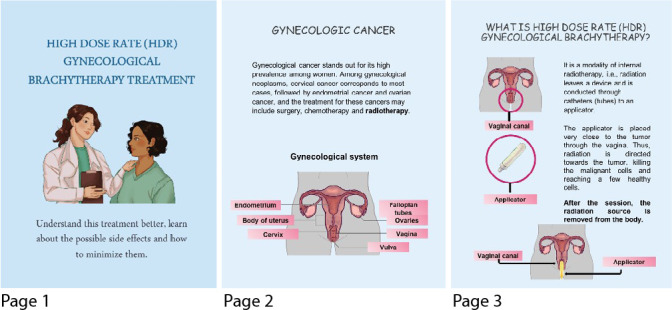
Some pages of the final version of the educational technology, Fortaleza, Ceará, Brazil, 2020

The completion of the two assessment rounds resulted in the final version of the educational technology. Figure 1 shows some pages of the EB.

## DISCUSSION

In the first version of the EB, the judges suggested approaching content, such as gynecological anatomy, in addition to a page on actinic cystitis. These suggestions were accepted, understanding that knowledge of one’s own body is relevant, as well as radiation-derived cystitis, which, although not very common, is a progressive and irreversible condition. The onset of this complication can be early, around six months, even years after the end of treatment, and the woman must be informed about the symptoms, to communicate to the health team and to avoid progression to more severe conditions^(19)^.

It is known that practices related to the management of side effects of gynecological BT, especially with regard to effects on sexuality, vary in treatment centers, with a decrease in attendance by women, due to side effects l^(20-21)^. In this regard, the effort of the multidisciplinary team to reinforce the attendance of patients is essential, in addition to minimizing the side effects, which are, in most cases, what hinder this constancy in HDR BT sessions.

Gynecological cancer is followed by well-documented psychosexual problems. At the same time, estimates of the incidence of suffering related to radiotherapy treatment are high in this population. Despite this, there are still few investigations of interventions to deal with these and other problems arising from treatment^(22)^.

In this way, having a booklet with guidelines based on scientific evidence and validated by experts can reduce the morbidity associated with the treatment, improving the quality of life (QoL) of these women and, consequently, increasing the attendance to treatment, which directly implies tumor control. This topic will be the subject of further investigation of this multi-method study.

Aiming to reduce the impact of pelvic RT in 82 women with gynecologic cancer and anal canal cancer, the clinical trial investigated sexual functioning and adherence to educational technology interventions at 3-, 6- and 12-months post-treatment. It was verified the understanding of the strategies and that the acceptability of the dilator to avoid vaginal stenosis was higher in the intervention group over the 12 months^(23)^. The authors recommend research using educational technologies for these patients. This shows that, even in the face of apparently embarrassing themes, women feel satisfied with the guidance provided, which can be a favorable resource in clinical practice.

It should be taken into account that the QoL of these women involves physical and socio-emotional aspects. Authors investigated the incidence of depression and QoL in this population, identifying higher levels in women with lower education level^(24)^. Similarly, a prospective cohort study, which recruited 90 post-treatment women for cervical cancer, assessed QoL, demonstrating that a factor that directly influences the QoL of this group is education, reinforcing the need for this guidance to patients. Other influencing factors in QoL were degree of tumor differentiation, tumor size, and tobacco use^(25)^.

On the use of vaginal showers or compresses with chamomile tea, recommended by only one judge, was not added to the EB. Although some health institutions advise on this practice^(26-27)^, no study with a good level of scientific evidence was identified to support this intervention.

The anti-inflammatory and healing results of aloe vera, documented in studies, refer, for the most part, to in vitro or animal research. The results in humans are old^(28-29)^ and conflicting, which can be partially explained, if we take into account the hydrophilic character of the components present in aloe vera, which are poorly permeable to skin barriers^(30)^.

A positive aspect in this study is due to the long experience in oncology of the judges, grouping knowledge involving oncology and educational technologies.

### Study limitations

Limitations include the difficulty in obtaining expert judges in the study area and the loss of judges between round 1 and 2 of Delphi.

### Contributions to nursing

The BT subspecialty still lacks studies that seek to improve nurses’ work, with a view to care based on scientific evidence. It is believed that the development of an educational technology can help nurses’ practice in the outpatient environment, in order to increase adherence to both treatment and guidelines discussed with the multidisciplinary team.

## CONCLUSIONS

The EB, constructed and validated by judges and women with gynecological cancer, resulted in an overall CVI equal to 0.98. The booklet construction involved, in addition to judges and patients, scientific and professional design knowledge. The printed version of the booklet is already being used in a public institution for women with gynecological cancer in Fortaleza/Ceará, where a clinical trial is being developed to assess the effectiveness of EB on HDR BT attendance, side effects of this treatment and level of anxiety.
